# Induction of prolonged natural lifespans in mice exposed to acoustic environmental enrichment

**DOI:** 10.1038/s41598-018-26302-x

**Published:** 2018-05-21

**Authors:** Yuichi Yamashita, Norie Kawai, Osamu Ueno, Yui Matsumoto, Tsutomu Oohashi, Manabu Honda

**Affiliations:** 1 Department of Functional Brain Research, National Institute of Neuroscience, National Centre of Neurology and Psychiatry, Kodaira, Japan; 2 Department of Research and Development, Foundation for Advancement of International Science, Tsukuba, Japan

## Abstract

We investigated the effect of acoustic environmental enrichment (EE) on the lifespans and behaviours of mice to the end of their natural lifespan in different acoustic environments. Acoustic EE induced a significantly prolonged natural lifespan (nearly 17% longer) and was associated with increased voluntary movements. However, no correlation between lifespan and voluntary movements was detected, suggesting that increased voluntary movements are not a primary cause of lifespan prolongation. Analyses of individual differences in lifespan demonstrated that lifespan extension induced by acoustic EE could be related to changes in social relationships (e.g., reduction of social conflict) among individuals kept within a cage. Therefore, an acoustic component may be an important factor inducing the positive effects of EE.

## Introduction

Environmental enrichment (EE) is a component of animal husbandry that aims to enhance sensory, cognitive, motor, and social stimuli, which are considered necessary for the optimal psychological and physiological well-being of animals. Many studies have demonstrated that EE induces various positive effects on experimental animals at multiple levels. For example, it has been shown to increase hippocampal neurogenesis^1,2^ and induce neural plasticity^3,4^. It also induces the acceleration of certain brain functions, including enhanced learning and memory^2,5^. Studies also showed that EE reduces the levels of anxiety/depression-like behaviour^6,7^. Moreover, improvements to the immune system and decreased oxidative-inflammatory stress have been reported in association with EE^6,7^.

Based on these observations of experimental animals, various studies have attempted to apply EE to human health care. These efforts have included physical, cognitive, and social enrichment as non-pharmacological interventions for cognitive impairment or psychiatric disorders^7–9^. However, relatively few studies of EE have been conducted on humans, and most published results have very low statistical power, rendering them inconclusive^9^.

The difficulty in conducting EE studies in humans mainly stems from the complexity of EE analyses. A standard EE experiment with animals involves the interaction of complex factors, including large cages with a variety of objects that enhance the animal’s physical activity and sensory stimulation, as well as a larger group of animals that are allowed to engage in social interactions^2,5^. Therefore, the effects of EE could be associated with these various factors. For example, increased physical activity (exercise), which is an expected result of environments with larger cages containing a variety of objects, is considered a critical component of EE^10–12^. Another important component of EE is social interaction^13,14^. For instance, a typical experimental EE setting uses larger groups of animals to enhance social interactions. The use of diverse sensory stimuli is another essential EE factor, with several studies demonstrating that enrichment with sensory stimuli in a particular modality has positive effects on the neural systems associated with the corresponding sensory modality^15–17^.

Our group has explored the physiological and behavioural effects of acoustic environmental information on humans and has demonstrated that acoustic EE induces diverse effects on humans^18–23^. We found that some natural environmental sounds, such as those of tropical rainforests, contain a wealth of inaudible high-frequency components (HFCs) above the range that is audible to humans^18^. Non-stationary sounds that contain significant quantities of HFCs evoke a significant increase in the regional cerebral blood flow in the midbrain and thalamus^19^ and in the occipital alpha frequency component of spontaneous electroencephalograms, compared with an otherwise identical sound from which the HFCs are removed^19–22^. In addition, the inclusion of HFCs renders the sound more pleasant to human listeners^19^, and listeners tend to spontaneously increase the comfortable listening level of the presented sound^19–21^. We call such phenomena collectively “the hypersonic effect”. The discovery of the hypersonic effect has strongly impacted the audio industry; cutting-edge digital audio media, such as Blu-ray discs and high-resolution audio, allow inaudible HFCs to be recorded. Acoustic EE utilizing the hypersonic effect was applied to improve urban acoustic environments^23^ and to induce clinical improvement in various psychiatric diseases^24^.

Acoustic EE has strong practical advantages. In particular, it can be introduced without manipulating any other environmental properties, does not require additional space or objects for physical exercise and visual stimulation, and does not require consideration of the optimal numbers of conspecific animals for social interactions. Thus, demonstrating the relevance of acoustic EE could provide an important contribution to the practice of EE.

We focused on the effects of acoustic environmental information as a single-factor EE in the present study, taking into account the possible future application to human studies. The relevance of acoustic EE as a single-factor EE was investigated by rearing mice in different acoustic environments to the end of their natural lifespan. The rearing conditions, other than the acoustic environment, were standard for experimental animals. Furthermore, based on our previous finding that the effects of acoustic EE on human are frequency dependent^19,22^, and even though audible ranges of sound differ between humans and rodents^25,26^, we also investigated the impact of frequency-dependent effects associated with acoustic EE.

## Results

### Experiment overview

The experimental animals were 96 8-week-old C57BL/6J mice assigned to three groups that were exposed to different acoustic environments for the duration of the experiment. These groups were (1) an acoustically enriched environment with a wide range of tropical rainforest sounds (~96 kHz, WRS group, n = 32, 16 males and 16 females); (2) an acoustically enriched environment with a narrow range of tropical rainforest sounds (~20 kHz, NRS group, n = 32, 16 males and 16 females); and (3) the control group, a standard environment without exposure to stimulation (CNT group, n = 32, 16 males and 16 females). Under the WRS and NRS conditions, tropical rainforest sounds were emitted through two speakers placed at the top of the cage during the day for the duration of the experiment. Tropical rainforest sounds were chosen on the basis of our previous findings, which indicated that these sounds contain the richest amount of high frequencies, along with complexity levels that induce acoustic EE effects in humans^18–23^. For the NRS condition, sound components above 20 kHz (the upper limit of the human audible range) were removed from the same sound source. Other environmental properties, including cage size, were standard for experimental mice. In all cases, the mice were housed in cages that each contained four animals of the same sex.

To observe the mice for their natural lifespan while causing them minimal stress, we avoided any invasive procedures, including blood sampling. The locations and movements of the animals in the cages were monitored throughout their lifespan using an infrared camera placed at the top of the cage. The shadows of animals in the field of view were detected as particles by real-time image processing. Voluntary movements were determined based on changes in the particle areas from time t to time t + 1. The particle numbers were recorded as an index of how much the animals formed aggregations in a cage. Because individual animals were not identified, the voluntary movements and the number of particles were calculated for each cage (i.e. a single value was determined for each cage). The interpretation of these cage-based measures was strongly dependent on the total number of animals in a cage. For example, a two-particle with four-animal measurement versus a two-particle with two-animal measurement was completely different significance. Therefore, in the current study, voluntary movements and particle numbers were analysed using only the data from the first 34 weeks of the experiment, when all animals were alive.

### Lifespan, voluntary movements, and particle number

**Figure 1:**
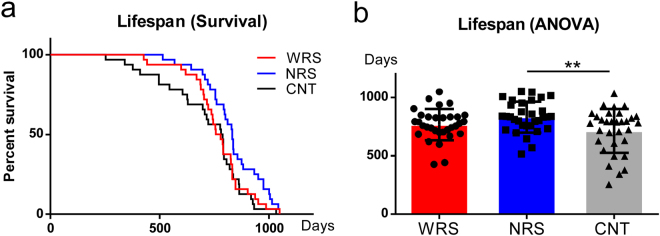
Lifespans for all sound conditions. (**a**) Survival curves and (**b**) average lifespan for all sound conditions: WRS (wide range of sounds), NRS (narrow range of sounds), and CNT (control). Error bars indicate the standard deviation.

Analysis of the lifespan survival curves using log-rank (Mantel-Cox) tests revealed a significant difference between the lifespans of mice under different sound conditions (*χ*^2^ = 6.64, *df* = 2, *p* < 0.05) (Fig. 1a). In addition, a one-way ANOVA was used to examine mean lifespans, and the results revealed a significant main effect of the sound conditions (*F*_*(2*,*93*)_ = 4.79, *p* < 0.05). Multiple comparisons revealed that the lifespans of mice exposed to the NRS condition were significantly longer than those of mice exposed to the CNT condition (*p* < 0.01) (Fig. 1b). Mice exposed to the WRS condition also had a longer lifespan than mice exposed to the CNT condition, but this effect was not statistically significant (*p* = 0.35).

**Figure 2:**
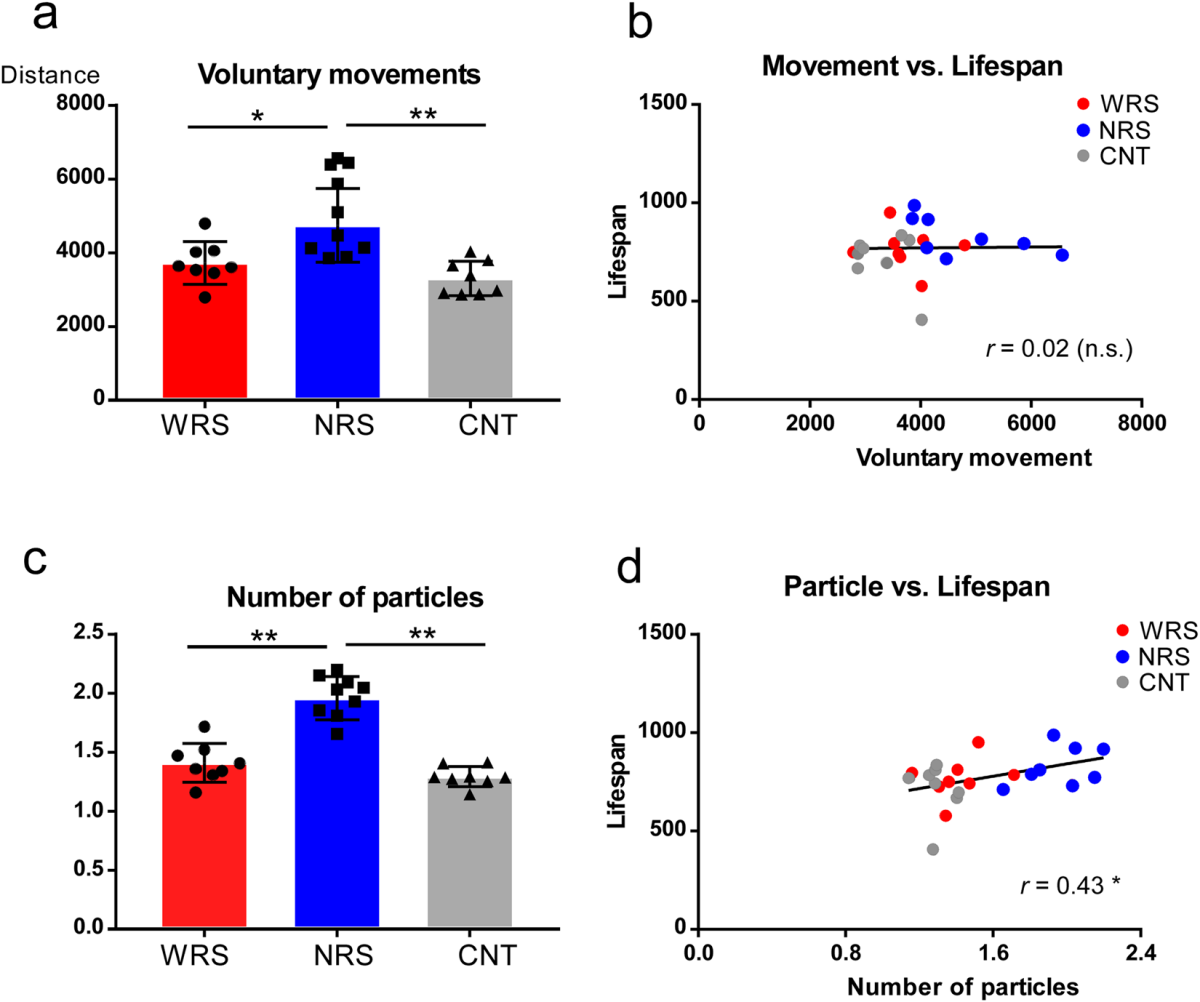
Voluntary movements, number of particles, and their relationship to lifespans for all sound conditions: WRS (wide range of sounds), NRS (narrow range of sounds), and CNT (control). (**a**) Mean voluntary movements and (**c**) number of particles for all sound conditions. Scatter plots for the functions of (**b**) voluntary movement and lifespan and (**d**) number of particles and lifespan. In the ANCOVAs of voluntary movement (**b**) and number of particles (**d**), average lifespan per cage was used, because voluntary movement and the number of particles were cage-based measures. Error bars indicate the standard deviation.

A one-way ANOVA was used to examine voluntary movements (1–34 weeks, the period when all animals were alive), and the results revealed a significant main effect of the sound conditions (*F*_*(*2,21*)*_ = 10.67, *p* < 0.01). Multiple comparisons revealed that the voluntary movements of mice exposed to NRS conditions were significantly greater than those of mice exposed to WRS (*p* < 0.01) and CNT (*p* < 0.01) conditions (Fig. 2a). Similarly, a one-way ANOVA was used to examine particle numbers (1–34 weeks), and the results revealed a significant main effect of sound conditions (*F*_*(2*,*2*1*)*_ = 45.15, *p* < 0.01). Multiple comparisons revealed that the particle numbers associated with mice exposed to the NRS condition were significantly larger than those of mice exposed to the WRS (*p* < 0.01) and CNT (*p* < 0.01) conditions (Fig. 2c). These results demonstrate that differences between sound environments affected the general health (lifespan) and behaviours (voluntary movements and positions of individuals in cages) of mice. There was no significant difference between mice exposed to the WRS and CNT conditions in terms of voluntary movement (*p* = 0.48) or particle numbers (*p* = 0.35).

Because exercise is considered as an important factor of EE, one could argue that a prolonged lifespan was the result of increased voluntary movements. However, there was no significant correlation between voluntary movements and lifespan (*r* = 0.02, *p* = 0.93*)* (Fig. 2b). Moreover, the results of the ANCOVA used to analyse the covariance between lifespan, voluntary movements, and sound conditions indicated a significant main effect of sound conditions (*F*_*(2*,*20)*_ = 4.03, *p* < 0.05), even after removing the effect of voluntary movements as a possible covariate factor. These results suggest that increased voluntary movements were not a primary cause of lifespan prolongation.

In comparison, there was a significant correlation between particle number and lifespan (*r* = 0.43, *p* < 0.05; Fig. 2d). Moreover, the results of the ANCOVA used to analyse covariance between the lifespan, particle number, and sound condition indicated that there was no significant main effect of sound condition after removing the effect of particle number (*F*_*(2*,*20)*_ = 0.19, *p* = 0.83). Because the particle number reflected the voluntary movements, there was a correlation between particle number and voluntary movements (*r* = 0.61, *p* < 0.01; Supplementary Fig. S1). However, this correlation cannot be explained solely by the increase in voluntary movements, because the particle number might reflect individual relationships within each cage.

### Sex and individual differences in lifespan

**Figure 3:**
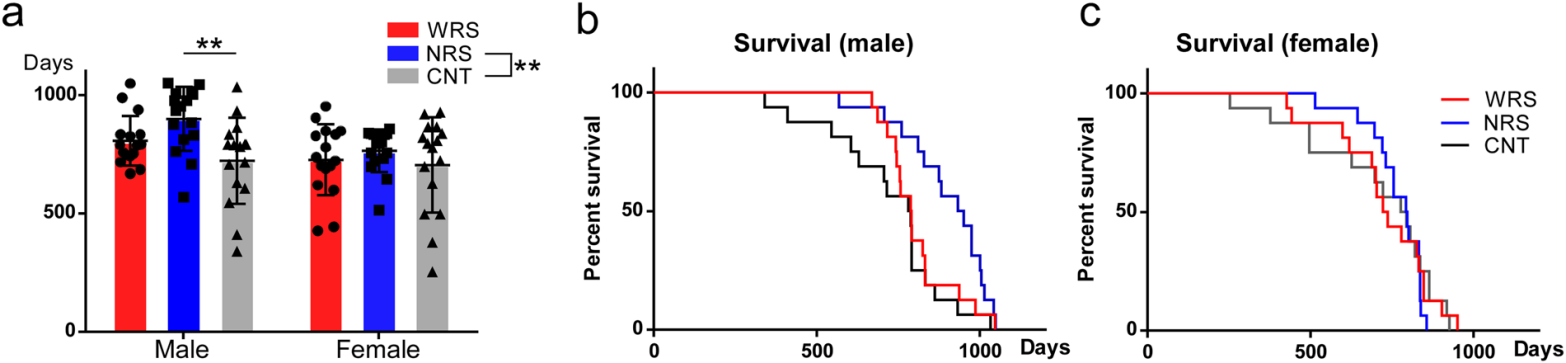
Lifespans of male and females for all sound conditions: WRS (wide range of sounds), NRS (narrow range of sounds), and CNT (control). (**a**) Average lifespans of male and females for all sound conditions. Lifespan survival curves for (**b**) males and (**c**) females. Error bars indicate the standard deviation.

Because the social relationships of individual mice are known to exhibit clear sex differences^27–29^, we further analysed lifespans focusing on sex differences. As shown in Fig. 3a, the average lifespan seems to be the longest under the NRS condition and the shortest under the CNT condition. This trend was similar in male and female mice, even though the prolongation of lifespan was more prominent in male mice. Two-way ANOVA of the lifespans for sex and sound condition variables revealed a significant main effect of sex (*F* (1, 90) = 6.45, *p* < 0.05) and sound conditions (*F* (2, 90) = 5.09, *p* < 0.01). However, there was no significant interaction between sex and sound conditions (*F* (2, 90) = 1.23, *p* = 0.30), suggesting that the lifespan prolongation induced by acoustic EE followed a similar trend for both male and female mice.

Multiple comparisons revealed that the lifespans were significantly longer under the NRS condition compared with that under the CNT condition (*p* < 0.01). We also separately analysed the average lifespans of male and female mice. One-way ANOVA of the male lifespans revealed a significant difference (*F* (2, 45) = 6.05, *p* < 0.01). Multiple comparisons revealed that the lifespans of males were significantly longer under the NRS condition than under the CNT condition (*p* < 0.01). Male mice exposed to the WRS condition also had a longer lifespan than mice exposed to the CNT condition, but this effect was not statistically significant (*p* = 0.23). Meanwhile, one-way ANOVA of female lifespans revealed no significant difference (*F* (2, 45) = 0.63, *p* = 0.54).

Similar trends were observed in lifespan survival curves of males and females (Fig. 3b,c). Separate analyses of the lifespan survival curves using a log-rank (Mantel-Cox) test indicated a significant difference in the lifespans of male mice exposed to different sound conditions (*χ*^*2*^ = 8.27, *df* = 2, *p* < 0.05) (Fig. 3b). In contrast, a separate analysis of the lifespan survival curves of females demonstrated no significant differences in the lifespans of mice exposed to different sound conditions (*χ*^*2*^ = 0.26, *df* = 2, *p* = 0.88) (Fig. 3c).

**Figure 4:**
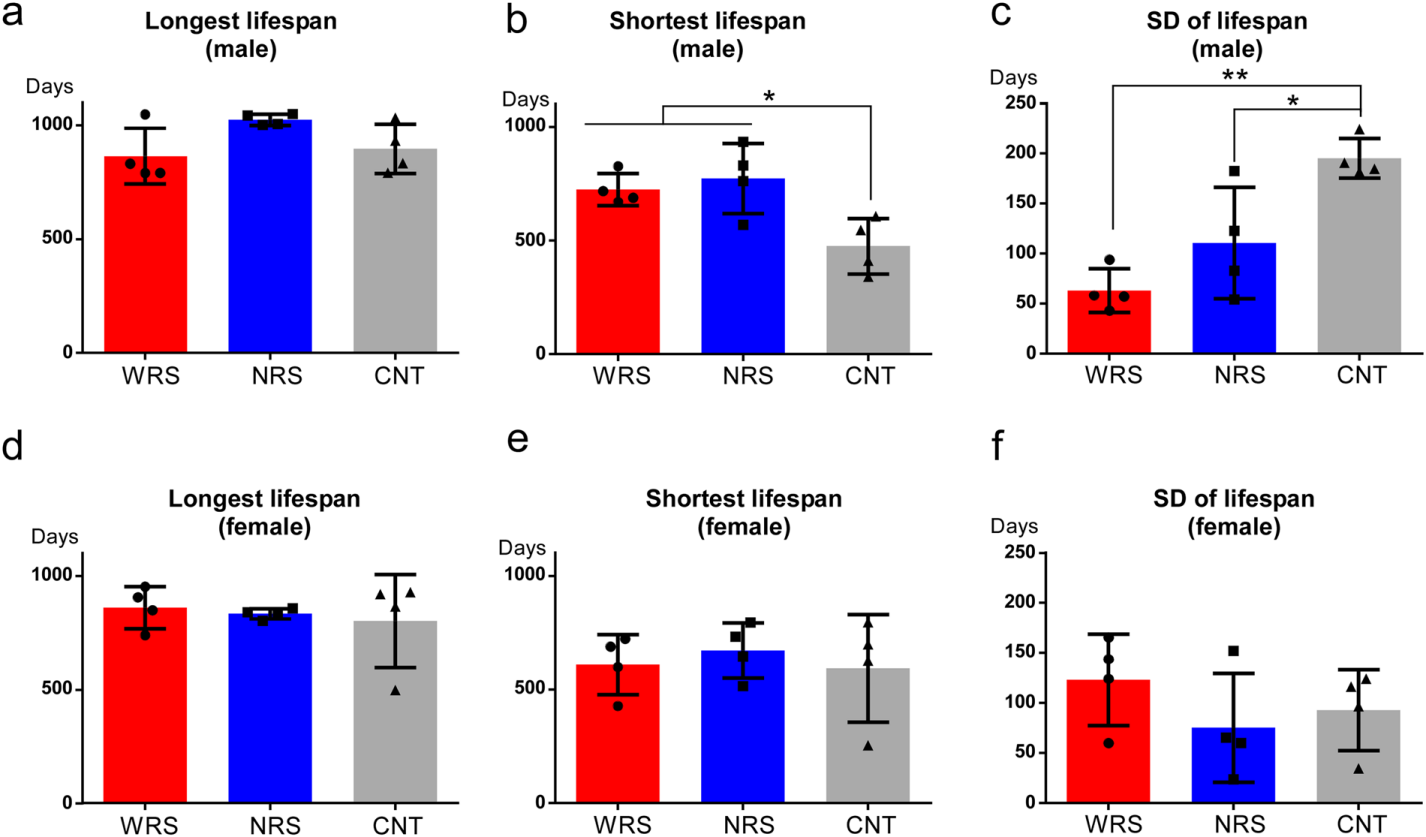
Individual differences in lifespan of each cage for all sound conditions: WRS (wide range of sounds), NRS (narrow range of sounds), and CNT (control). Longest lifespans of (**a**) males and (**d**) females. Shortest lifespans of (**b**) males and (**e**) females. Standard deviation (SD) of lifespans of (**c**) males and (**f**) females. ANOVAs of the longest lifespan, shortest lifespan, and SD of lifespan were conducted using representative values for each cage calculated on the basis of individual lifespans. Error bars indicate the standard deviation.

Based on the survival curves, the longest lifespans did not differ between sound conditions, suggesting that the statistical difference in lifespan was primarily the result of differences between the lifespans of individuals, rather than being affected by mice with the longest lifespans. One possible factor affecting the lifespans of experimental animals is social hierarchy. To address this issue, we conducted more detailed analyses that focused on the differences in the lifespans of different individuals within each cage. One-way ANOVA tests used to analyse the longest lifespans, shortest lifespans, and variation in lifespans (standard deviation: SD) within each cage for each sex were conducted for sound condition variables. In male mice, the sound condition had a significant main effect on the shortest lifespans (*F*_*(2*,*9)*_ = 7.06, *p* < 0.05, Fig. 4b) and the SD of lifespans (*F*_*(2*,*9)*_ = 13.62, *p* < 0.01, Fig. 4c). Multiple comparisons revealed that the shortest lifespan of male mice under the CNT condition was significantly shorter than those of mice under the WRS (*p* < 0.01) and NRS (*p* < 0.05) conditions (Fig. 4b). Furthermore, the SD of male lifespans exposed to the CNT condition was significantly greater than those of males exposed to the WRS (*p* < 0.01) and NRS (*p* < 0.05) conditions (Fig. 4c). Meanwhile, in female mice, there were no significant differences in the longest lifespans (*F*_*(2*,*9)*_ = *0.20*, *p* = *0.82*, Fig. 4d), shortest lifespans (*F*_*(2*,*9)*_ = *0.23*, *p* = *0.80*, Fig. 4e), or SD of lifespans (*F*_*(2*,*9)*_ = *1.06*, *p* = *0.39*, Fig. 4f).

## Discussion

The present study demonstrates that mice exposed to the NRS condition had significantly longer lifespans (nearly 17% longer) than those exposed to the CNT condition. This is the first study to show that a prolongation of the natural lifespan of mice can be induced solely by auditory sensory enrichment, adding to previous work that showed that EE with complex factors can induce a prolongation of the natural lifespan^6,30^. In addition, the results demonstrate that voluntary movements increased significantly under the NRS condition, compared with the CNT condition. Even though an increase in physical movement is considered an important factor of EE, additional analyses of the relationship between voluntary movement and lifespan suggest that increased voluntary movement is not a primary cause of lifespan prolongation. This result is consistent with recent findings showing that spontaneous exercise does not necessarily prolong lifespan^31^, even though physical exercise might prevent several signs of frailty, including decreased strength, endurance, and motor coordination associated with increased mitochondrial biogenesis in skeletal muscles and cortical brain derived neurotrophic factor levels^32^. The results also demonstrate that the particle numbers measured in mice exposed to the NRS condition were significantly greater than those of mice exposed to the CNT condition. Although particle number was correlated with voluntary movements (Fig. S1), an increase in the number of particles might reflect changes in the relationships between individuals within the cage.

Additional analysis of two-way ANOVAs of the lifespans for sex and sound condition variables demonstrated that the prolongation of lifespan induced by acoustic EE showed similar trends for both male and female mice, even though the prolongation of lifespan was more prominent in male mice. One possible explanation for this sex difference in the effects of acoustic EE on lifespan is sex differences in the social relationships of mice. When mice were housed in a group, social hierarchy or dominant-submissive relationships are invariably observed in both male and female mice, although to a lesser extent in female mice^29^. Social hierarchy ranking is known to be related to the physical and psychological health of animals; submissive and low-ranking animals were observed to suffer from social stress, resulting in a higher rate of viral infections and tumour formation^33,34^. Thus, one can expect that as the social hierarchy in a cage strengthens, there will be a greater lifespan discrepancy between individuals with minimum and maximum lifespans (i.e. lifespan variance in a cage). Indeed, we observed significant sex differences in individual lifespans within each cage. In male mice, the minimum lifespan in each cage was significantly prolonged under the WRS and NRS conditions compared with that under the CNT condition. In addition, lifespan variation within each cage was significantly smaller under the WRS and NRS conditions than under the CNT condition. In female mice, however, there were no significant differences in the longest lifespans, shortest lifespans, or the variation of lifespans. These observations suggest that social conflict may be reduced when acoustic EE is present, and that this effect is more prominent in males due to the higher levels of social conflict that they experience.

Regarding sound frequency–dependent effects, changes in lifespan, voluntary movement, and particle number were mainly observed under the NRS condition for both males and females. In comparison, additional analyses of lifespan, including lifespan variation in the cage, suggest that both WRS and NRS sounds had similar effects on males, but not females. These observations suggest that the WRS and NRS conditions have similar primary effects on mice, but that the high-frequency component of WRS might, somehow, negate the effects of acoustic EE. Although the present study did not provide sufficient evidence, we speculate that the high-frequency component of WRS might disturb communications in mice using ultrasonic vocalization, which is dominant in female mice. The current observation of sex difference suggests that the frequency-dependent effects of acoustic EE may be related to sex differences in ultrasonic vocalization in mice^35–37^, again consistent with attenuated effects of acoustic EE on the prolongation of lifespan in female mice.

The relationship between the audible ranges of sounds and the frequency-dependent effects of acoustic EE is an important issue for the possible application of acoustic EE in humans. Our results demonstrated that changes in lifespan and behaviour of mice were mainly observed under the NRS condition, which corresponds to the range that is audible to humans. On the contrary, previous studies in humans reported that sounds containing inaudible high-frequency components can induce diverse positive effects^19–24^. One possible explanation for this discrepancy is that the optimal range of sound frequency for acoustic EE may differ from the range used by humans for vocal communications. In other words, high-frequency sounds that are inaudible to humans and out of the range of human vocal communication can work as acoustic EE for humans, while lower frequency sounds that are audible to humans but are out of the range of vocal communication in mice can work as acoustic EE for mice. However, even within the range of high-frequency sounds that is inaudible to humans, these effects of acoustic EE show frequency dependence: 16–32 kHz sounds induced a negative effect (reduced alpha power in electroencephalograms), while sounds above 32 kHz induced positive effects (increased alpha power in electroencephalograms)^22^. Moreover, these physical frequency-dependent effects are similar to the use of frequency ranges of ultrasonic vocalizations of rodents; rats use lower frequency bands (around 20 kHz) to express negative emotions and higher frequency bands (around 50 kHz) for positive emotions^38^. Therefore, it remains possible that physical frequency itself is an important factor in acoustic EE.

We avoided using any invasive procedures, including blood sampling, to measure natural lifespan and observed general behaviour and lifespan alone. Therefore, the present study did not provide sufficient evidence on the biological mechanisms underlying the prolongation of lifespans by acoustic EE. Despite this limitation, the present study may provide an important working hypothesis in the field of EE. For example, we propose a testable hypothesis for the effects of acoustic EE (i.e. that changes in the social relationships of mice are induced by acoustic EE). Moreover, changes in the voluntary movements and particle number were observed during the early stages of the experiments (e.g. 2–4 weeks after intervention) (Fig. S2). Thus, we suggest that shorter term interventions of acoustic EE that measure social behaviours and physiological changes related to psychological stress and healthy aging, including the levels of stress hormones and agents related to healthy aging, could be an effective approach to examine this hypothesis.

## Methods

### Animals

The study animals were 96 8-week-old C57BL/6J mice assigned to three groups with different acoustic environments for the duration of the experiment. Each group consisted of 32 animals (16 males and 16 females). In all the sound conditions, mice were housed as groups in cages (172 × 240 × 129 mm) that each held four animals. All mice were placed on a 12-hour light cycle beginning at 20:00. The temperature and humidity of the cages were maintained at approximately 24 °C and 40–60%, respectively. Food and water were fed ad libitum. All experiments were approved and conformed to the guidelines set by the Small Animal Welfare and Ethics Committee of the National Institute of Neuroscience, National Center of Neurology and Psychiatry, Kodaira, Japan.

### Sound stimulations

**Figure 5:**
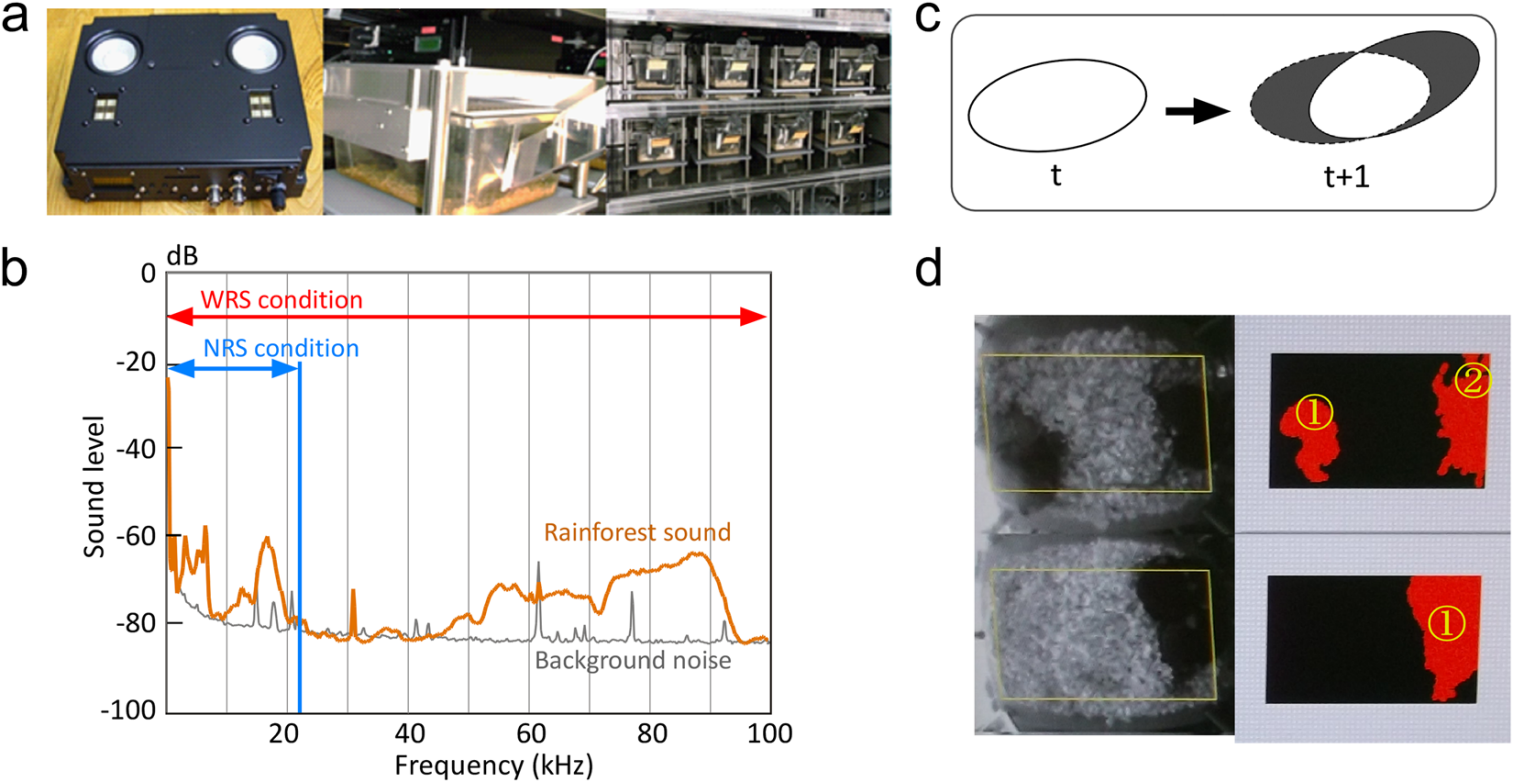
Experimental settings and behaviour measures. (**a**) Sound emission system fixed at the top of each cage. (**b**) Power spectrum of sounds emitted through the speakers for WRS (wide range of sounds) and NRS (narrow range of sounds) conditions. Orange and grey lines indicate the frequency power spectra of rainforest sounds and background noise, respectively. (**c**) Measurements of voluntary movement and the particle number. Shadows of animals in the field of view were detected as particles. Voluntary movement was determined as exclusive or (exclusive disjunction) as particle areas from time *t* to time *t* + *1*. (**d**) The number of particles was recorded as an index of the degree of animals forming aggregations. Voluntary movement and particle number were monitored for each cage separately.

Under the WRS and NRS conditions, tropical rainforest sounds were emitted through two speakers fixed at the top of the cage (Fig. 5a) throughout the day, for the duration of the experiment (until the end of the natural lifespan). Tropical rainforest sounds containing rich amounts of high frequencies were chosen as the sound source for the acoustically enriched environment, based on our previous findings which indicated that the sounds richest in high frequencies induced acoustic EE effects in humans^18–23^. For the NRS condition, sound components above 20 kHz (the upper limit of the range audible to humans) were removed from the same sound source (Fig. 5b). This approach was based on our previous findings from experiments in humans, which indicated that acoustic EE effects are frequency dependent^19,22^.

### Lifespan

Individual lifespan was used in the survival curve analysis and ANOVAs of average lifespans under the different sound conditions. In the ANCOVAs of voluntary movements and particle numbers, cage-based-averaged lifespan was used, because voluntary movements and particle numbers were cage-based measures. In the analysis of the longest lifespans, shortest lifespans, and SD of lifespans, ANOVAs were conducted using a representative value for each cage and were calculated based on individual lifespans. To measure natural lifespans, we avoided any invasive procedures, including blood sampling.

### Voluntary movement and particle number

The location and movement of animals in cages were monitored using an infrared camera located at the top of the cage. This camera recorded footage throughout the lifespan of the animals. The infrared camera images were sampled every 500 ms, and shadows of animals in the field of view were detected as particles using real-time image processing (Fig. 5d). Voluntary movements were determined as exclusive or (exclusive disjunction) from particle areas from time *t* to time *t* + *1* (Fig. 5c). Because individual animals were not identified, voluntary movements were calculated as a single value for each cage every 500 ms. The number of particles was recorded as an index of how much the animals formed aggregations. Particle number was monitored for each cage separately.

### Data analysis

Statistical analyses of the lifespan survival curves were performed using a log-rank (Mantel-Cox) test. Analyses of the mean values of lifespan, voluntary movement, and particle number were performed using ANOVA tests. Regarding the lifespan, ANCOVA tests were also conducted using voluntary movement and particle number as covariates. All post-hoc multiple comparison tests were performed using Shaffe’s modified sequentially rejective Bonferroni procedure, and the level of statistical significance was set at *p* < 0.05.

## Electronic supplementary material

Supplementary information
