# Supplementary material for: Induction of prolonged natural lifespans in mice exposed to acoustic environmental enrichment
Source: Sci Rep. 2018 May 21;8:7909. doi: 10.1038/s41598-018-26302-x (PMC5962611; doi:10.1038/s41598-018-26302-x)
Supplement: Supplementary file 1 — Supplementary information [file 41598_2018_26302_MOESM1_ESM.pdf]

Supplementary Information for
'Induction of prolonged natural lifespans in mice exposed to acoustic environmental enrichment'

by Yuichi Yamashita, Norie Kawai, Osamu Ueno, Yui Matsumoto, and Tsutomu Oohashi, and Manabu Honda

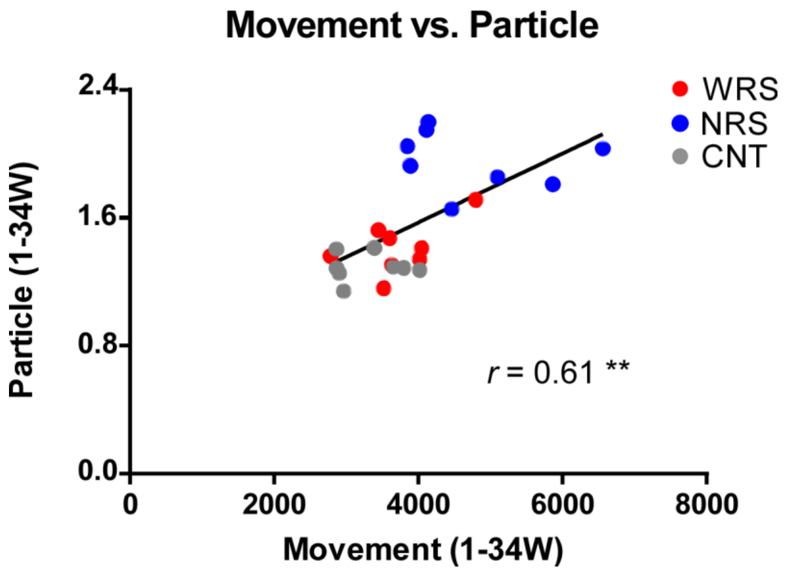

Figure S1. Scatter plots of the functions of voluntary movement and number of particles, for the WRS (wide range of sounds), NRS (narrow range of sounds), and CNT (control) conditions.

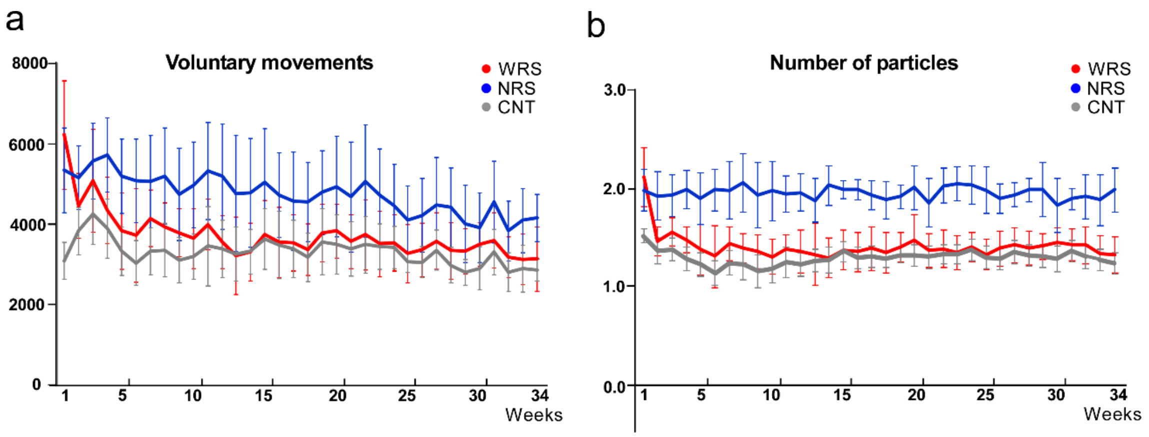

Figure S2. Changes to voluntary movement and number of particles over time for all sound conditions: WRS (wide range of sounds), NRS (narrow range of sounds), and CNT (control). The plotted data were collected from the first to the 34th week of the experiment (during which time all animals were alive). Error bars indicate the standard deviation.
